# Assessing Thai Hospitals’ Evacuation Preparedness Using the Flexible Surge Capacity Concept and Its Collaborative Tool

**DOI:** 10.1007/s13753-023-00468-z

**Published:** 2023-02-15

**Authors:** Phatthranit Phattharapornjaroen, Eric Carlström, Lina Dahlén Holmqvist, Yuwares Sittichanbuncha, Amir Khorram-Manesh

**Affiliations:** 1 Institute of Clinical Sciences, Department of Surgery, Sahlgrenska Academy, University of Gothenburg, 40530 Gothenburg, Sweden; 2 Department of Emergency Medicine, Faculty of Medicine Ramathibodi Hospital, Mahidol University, Bangkok, 10400 Thailand; 3 Institute of Health and Care Sciences, Sahlgrenska Academy, University of Gothenburg, 40100 Gothenburg, Sweden; 4 Gothenburg Emergency Medicine Research Group, Sahlgrenska Academy, University of Gothenburg, 40530 Gothenburg, Sweden; 5 USN School of Business, University of South-Eastern Norway, 3603 Kongsberg, Norway; 6 Institute of Medicine, Department of Internal Medicine and Clinical Nutrition, Sahlgrenska University Hospital, 40530 Gothenburg, Sweden

**Keywords:** Collaborative tool, Disaster preparedness, Flexible surge capacity, Hospital evacuation, Multiagency collaboration, Thailand

## Abstract

According to the concept of “flexible surge capacity,” hospitals may need to be evacuated on two occasions: (1) when they are exposed to danger, such as in war; and (2) when they are contaminated, such as during the Covid-19 pandemic. In the former, the entire hospital must be evacuated, while in the latter, the hospital becomes a pandemic center necessitating the transfer of its non-contaminated staff, patients, and routine activities to other facilities. Such occasions involve several degrees of evacuation—partial or total—yet all require deliberate surge planning and collaboration with diverse authorities. This study aimed to investigate the extent of hospital evacuation preparedness in Thailand, using the main elements of the flexible surge capacity concept. A mixed method cross-sectional study was conducted using a hospital evacuation questionnaire from a previously published multinational hospital evacuation study. The tool contained questions regarding evacuation preparedness encompassing surge capacity and collaborative elements and an open-ended inquiry to grasp potential perspectives. All 143 secondary care, tertiary care, and university hospitals received the questionnaire; 43 hospitals provided responses. The findings indicate glitches in evacuation protocols, particularly triage systems, the inadequacies of surge planning and multiagency collaboration, and knowledge limitations in community capabilities. In conclusion, the applications of the essential components of flexible surge capacity allow the assessment of hospital preparedness and facilitate the evaluation of guidelines and instructions through scenario-based training exercises.

## Introduction

Hospitals play a crucial role in disasters caused by natural hazards, manmade threats, and public health emergencies (DPHE), aiming to optimize the outcomes of medical management of injuries and to reduce the suffering of victims under limited access to resources (Khorram-Manesh and Burkle 2020; CRED and UNDRR 2020). Some emergencies directly affect hospitals, resulting in partial or total evacuation—for example, natural hazards such as hurricanes destroy several hospital structures in the United States annually (Mattox 2006; Griffin et al. 2019; Hines and Reid 2021); flooding in the middle of Thailand affected hospitals causing infrastructure destruction and interrupting patient care (Khorram-Manesh et al. 2014); and in the ongoing Ukrainian war, hospitals and other vital civilian service sectors are frequently attacked, resulting in the deaths and injuries of civilians living in or adjacent to the targeted areas (Al Jazeera 2022). Other events may indirectly impact a hospital—for example, a pandemic, such as the still-active Coronavirus 2019 (Covid-19) infection, which prevents unaffected hospitals from admitting new patients, and forces affected hospitals to partially or entirely evacuate unaffected patients and their routine activities. In both cases, there is also a need to isolate out-of-hospital cases and offer care at home using potential diagnostic and surveillance methods (Phattharapornjaroen et al. 2022b). All these events that necessitate partial or total evacuation require extra resources and an expansion of healthcare capacity through multiagency collaboration. The capacity expansions to multiple agencies require synchronized and tested collaboration, preferably using collaborative elements to harmonize the working process, developing from coordination (that is, resource-pooling and adapting), cooperation (that is, distribution of tasks in an agreed order), communication (that is, information sharing), to collaboration (that is, achieving the same goals) (Gajda 2004; Lozano et al. 2021).

Hospital evacuation is defined by the United Nations Office for Disaster Risk Reduction (UNDRR) as a process of “Moving people and assets temporarily to safer places before, during or after the occurrence of a hazardous event to protect them” (UNDRR 2009). The UNDRR expresses the complex chain of collaboration needed to achieve the goals over an extended period. The Pan American Health Organization (PAHO) focuses on the goal of evacuation that is to safeguard the health and lives of its occupants (Pan American Health Organization 2018). This indicates the need for protection of both patients and medical staff and indirectly creates moral and ethical issues of whom to evacuate and who has the power to decide. Other researchers, for example, Tekin et al. (2017), emphasized an attempt to empty an entire hospital or a part of it as the description of a hospital evacuation, which ultimately presents different aspects of evacuation, that is, where should the occupants of a hospital be moved to if totally evacuated.

Irrespective of definitions, evacuation inherently increases the risks for patients, staff, and other people nearby when transferred due to a variety of internal and external factors that may impact them (Tekin et al. 2017). Moreover, hospitals serve a large number of people who require special assistance, such as those with physical impairments or hearing and vision difficulties, which complicates the evacuation process (Al-Wathinani et al. 2022). Within this complex process, there is a need for critical decision making in several levels of action and care. The decision to evacuate frequently arises from safety threats or total operation disruptions with eminent impact on infrastructure (McGinty et al. 2016; Ariscain 2019). Decision making is challenging and requires the ability to command and control the necessary activities in several lines of command. Reasonable control of the situation and collaboration with other agencies necessitate appropriate communication ability and updated risk and vulnerability assessments to continuously plan and implement new measures, including what is needed to guarantee patient safety, triage, treatment, and transport to the proper medical and non-medical facilities (Wabo et al. 2012; Hicks and Glick 2015). The surge planning includes four essential elements (4S): (1) Staff comprising both medical and non-medical personnel; (2) Stuff such as medical devices and ambulances; (3) Structure or Spaces that refer to needed areas to be modified to either treatment zones or shelters; and (4) Systems that refer to practical or mutual guidelines (Bonnett et al. 2007; Hick et al. 2014). Additionally, the multiagency approach to disaster and emergency response requires a collaborative instrument to harmonize the interaction between agencies (Sammut et al. 2001; Bahrami et al. 2020; Khorram-Manesh and Burkle 2020; Phattharapornjaaroen et al. 2022a, 2022b).

**Fig. 1:**
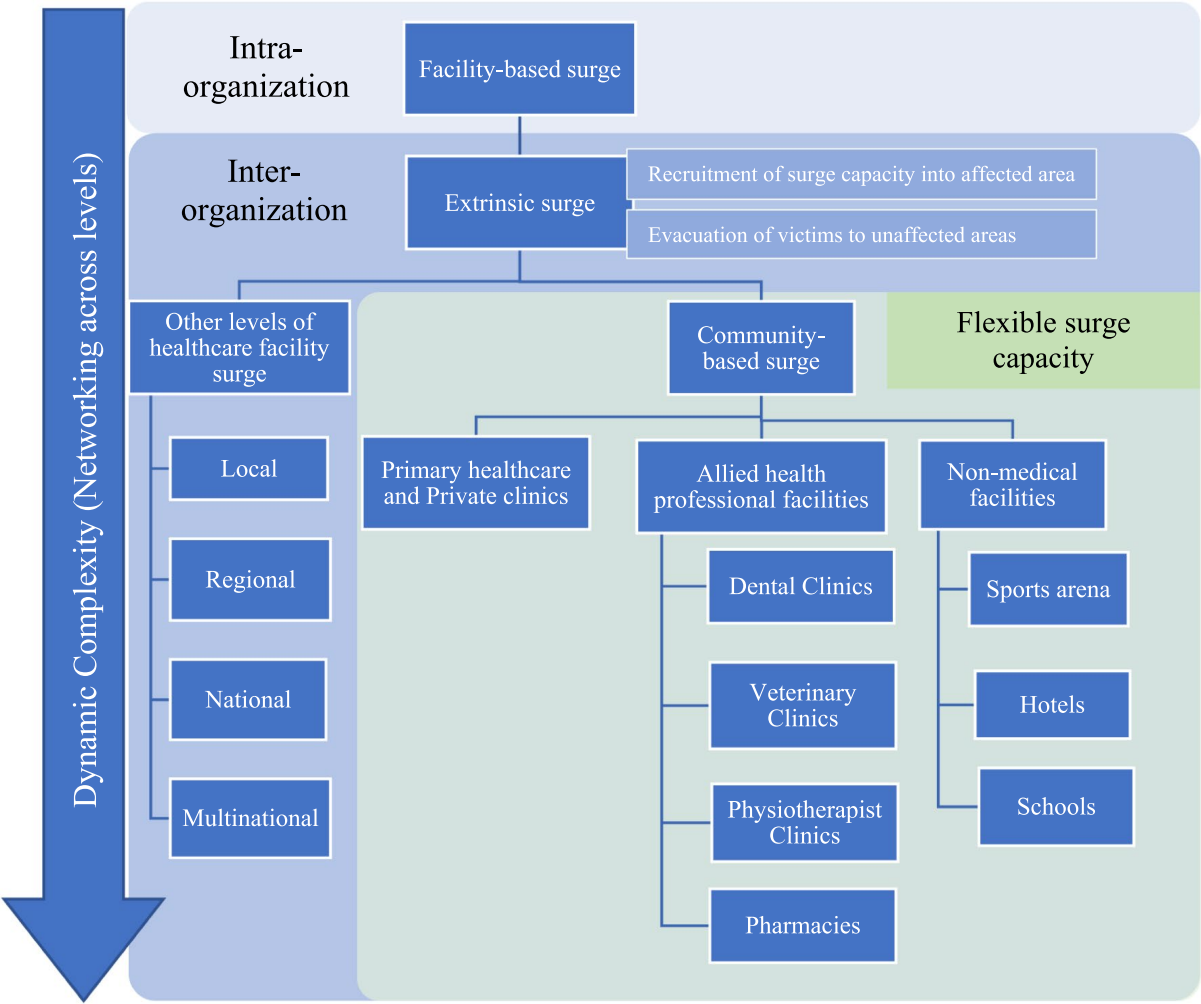
Surge capacity framework incorporating the flexible surge capacity concept. *Source* Phattharapornjaroen et al. (2022a)

One of the approaches to expanding the response capacity is through the flexible surge capacity (FSC) concept, in which the collaboration is extended further to embrace communities’ potential resources and facilities (Khorram-Manesh 2020; Phattharapornjaroen et al. 2022b). According to the FSC theoretical framework, community resources can be utilized when hospitals are the target of a hazardous event and cannot receive more patients,for example, under explosion threats, during a pandemic where the restriction policy is applied, or when the hospitals are overloaded with ordinary and disaster-related patients (Fig. 1). In such scenarios, and when other medical resources are not accessible or available, local resources, such as primary care, veterinary, and dental clinics may contribute their staff, stuff, and spaces, while hotels, schools, and sports halls may provide food, space for caring for light injured, and other appropriate needs (Glantz et al. 2020; Phattharapornjaroen et al. 2021, 2022a). A key element in organizing such a process is to create a system that governs the collaboration between these organizations and facilities (Yazdani et al. 2021; Khorram-Manesh, Dulebenets, et al. 2021). Such a system needs a collaborative tool to enhance interactions between agencies and organizations. To facilitate interactions, FSC uses the elements in Major Incident Medical Management and Support (MIMMS) education—the CSCATTT, standing for Command and control, Safety, Communication, Assessment, Triage, Treatment, and Transport—to organize the procedures to be conducted and create interaction between different agencies (Sammut et al. 2001; Hicks and Glick 2015). During an evacuation, hospitals require substantial support to transfer patients and victims, devices, and medical supplies safely and systematically. The CSCATTT can, thus, create flexibility between the most common partners in such events, that is, healthcare, rescue teams, and police forces, to achieve the goals for each element of the CSCATTT (McGinty et al. 2016; Mace and Sharma 2020; Yazdani et al. 2021).

Khorram-Manesh, Phattharapornjaroen, et al. (2021) reported the current perspectives on hospital evacuation in 15 countries using CSCATTT as a collaborative tool. They showed that most countries lacked the necessary collaborative elements and readiness for evacuation; some even lacked an evacuation plan. Despite several reports and changes in the national preparedness system, the necessary preparedness for management and response to diverse emergencies, particularly in hospital evacuations, is missing (Khorram-Manesh, Phattharapornjaroen, et al. 2021; Yazdani et al. 2021). The paucity of readiness was evident in the devastating 2004 Southeast Asian Tsunami, which caused numerous casualties and deaths (Wattanawaitunechai et al. 2005). Additionally, other incidents, such as flooding in Thailand in 2011 and the Covid-19 pandemic, have illustrated insufficiencies in several parts of the response system, including command and control, maintenance, and logistics (Khorram-Manesh et al. 2014; Dejchaisri 2019; Nittayasoot et al. 2021; Phattharapornjaroen et al. 2022b). In both events, the need for partial or total hospital evacuation in affected areas was eminent. However, there has been no evident improvement in producing guidelines or educational initiatives (Khorram-Manesh, Dulebenets, et al. 2021; Al-Wathinani et al. 2022). The FSC concept may, however, facilitate the evacuation processes and exercise initiatives to enhance the multiagency and multi-professional synchronization and collaboration needed in such an event (Phattharapornjaroen et al. 2022a).

Thailand is a disaster-prone country, where natural hazards such as flooding, manmade hazards such as protests and conflicts, and public health emergencies such as pandemics continuously increase with clear impacts on the Thai society (Peltz et al. 2006; Angthong et al. 2012; Khorram-Manesh et al. 2014; Nittayasoot et al. 2021). Thai public healthcare facilities, responsible for all citizens’ health, provide care to a geographically defined population divided into 12 regions with similar cultures, risks, and environments. The facilities have four levels of competencies and capacities: primary care, secondary care, tertiary care, and university hospital. Additionally, private hospitals’ capabilities are comparable to the public tertiary care tiers, and they are potential candidates to respond hand in hand with public and community facilities. In a previous study, community resources were used during the Covid-19 pandemic in Bangkok, and the concept of FSC could successfully be implemented (Phattharapornjaroen et al. 2022b). Although the diversity of the Thai hospital system can be a drawback (Leerapan et al. 2021), it still might be suitable for implementing the FSC concept and enhancing the community response system in other situations described by FSC, that is, during hospital evacuation. This study explored Thai hospitals’ current evacuation readiness and preparation regarding surge capacity and collaboration, using the concept of FSC and its collaborative tool. A successful assessment may be used to evaluate hospital preparedness and as a tool for future educational initiatives.

## Method

A mixed method cross-sectional study was performed employing an adapted hospital evacuation questionnaire to assess evacuation preparedness of hospitals using the FSC concept and collaborative tools.

### Study Participants and Procedure

The study focused on the secondary care (120−500 beds capacity), tertiary care (500 beds capacity), and university hospital (400−2265 beds capacity) levels because these levels of hospital serve complex conditions and comprise specialized healthcare staff, modern medical devices, procedures, prompted areas for treatments, and up-to-date guidelines and protocols (Tangcharoensathien et al. 2018; Tejativaddhana et al. 2018). These characteristics are relevant to major incidents and disaster responses. The names and information of a total of 143 hospitals were obtained from the Ministry of Health of Thailand.

Initially, the research team phoned the hospital administrations to communicate the study’s purposes, details, and requirements and let them purposively select their representatives to participate in the survey. These representatives knew the hospital’s emergency response plans and protocols, possessed the authority to revise them, and were responsible for communicating with the disaster preparedness committee on potential issues he/she could not decide for. They could be the facility’s director, safety officer, head of the emergency department, or one with an equivalent role. When the representatives were selected, they were contacted to verify their positions and ensure voluntary participation. The formal letter containing research details, and an online link for consent and the questionnaire were then sent to the participants through authorized hospital contact details. Following the letters’ deliveries, participants were approached four times by administrative emails and phone calls to be reminded of the study. The respondents’ notifications were stopped after the preliminary analysis was conducted, and the qualitative data were saturated by repetitions and similarities of the findings.

#### Study Tool^1^

The hospital evacuation questionnaire used in this study was adapted from a previously published multinational hospital evacuation study (Khorram-Manesh, Phattharapornjaroen et al. 2021). The questionnaire was posted on the Google Form Platform and was available for responses from December 2021 to April 2022. The tool was created using the nominal group technique in several rounds by three independent expert reviewers based on the literature review between 2002 and 2018 and the content analysis. The tool was then validated with face and content validity. It contained relevant questions about hospital evacuations’ responses and preparedness encompassing elements in surge capacity (4S) and collaboration (CSCATTT) as well as ethical and legal issues related to preparedness, situation assessment, and management of vulnerable groups. Additionally, the final open-ended question was designed to collect possible perspectives and comments. The English version of the questionnaire was translated into the native language by two native Thai speakers independently and then back-translated into English. Subsequent face validation and a comparison to the original version were performed, and consensus regarding differences in wording and meaning was reached through discussion between translators who were emergency field professionals. Finally, to ensure the contents’ accuracy and coherence, the documentation of the translation process was presented and discussed with the original developer. Once the translation process was complete, the questionnaire, research details, consent, and ethical approval declaration were transferred to an online form (Ball 2019).

### Data Collection and Analysis

All responses were transferred from the Google Form to Google Sheets and Microsoft Office EXCEL.^1^ The hospital’s bed capacity, locations, and details in 4S and CSCATTT were recorded. For quantitative data, statistical calculations were performed using Stata Version 17 software. The data were imported into Stata 17. The descriptive data were presented in counts and proportions. The association between hospitals’ bed capacity and elements of surge capacity and collaboration were compared using Chi-square or Fisher’s exact test (Fisher’s exact test was used if the expected number in each cell were below 5). The statistical significance was set at 5% (p < 0.05).

The qualitative data from open-ended responses were analyzed using content analysis. The accumulated data were read several times, then coded and grouped for themes. A content analysis was conducted (Hsieh and Shannon 2005) based on the elements of surge capacity (4S) and the collaborative elements (CSCATTT), which were evaluated and elaborated as part of the FSC concept (Glantz et al. 2020; Khorram-Manesh 2020; Phattharapornjaroen et al. 2021; Phattharapornjaroen et al. 2022a). All coded and grouped data were discussed until reaching a consensus among authors.

## Results

Of the 143 hospitals, 43 hospitals from different parts of Thailand answered the survey and provided their contingency plans in attached files to confirm their responded information. The characteristics of responding hospitals are shown in Table 1. There were no statistically significant associations between the hospital’s bed capacity and elements of surge capacity and collaboration.

**Table 1:** Characteristics of the 43 surveyed Thai hospitals

Functions	No. of Hospitals (%)
Geographical locations	
Northern	5 (11.6)
Northeastern	11 (25.6)
Eastern	3 (7.0)
Western	2 (4.7)
Middle	13 (30.2)
Southern	9 (20.9)
Beds capacity	
More than 1000 beds	3 (7.0)
501−1000 beds	19 (44.2)
300−500 beds	9 (20.9)
< 300 beds	12 (27.9)

### Surge Capacity

The Table 2 shows respondents’ surge planning and other preparedness for hospital evacuation.

**Table 2:** Preparedness regarding hospital evacuation in 43 Thai hospitals

Functions	No. of Hospitals (%)
Incident command system	43 (100)
Hospital act independently	42 (97.6)
Surge capacity	
Staff	43 (100)
Stuff	36 (83.7)
Structure	27 (62.8)
System	29 (67.4)
Management plan for vulnerable patients/relatives/staff	15 (34.9)
Plan for critical transportation	28 (65.1)
Ethical agreement in a hospital evacuation plan	
Official	3 (7.0)
Unofficial	13 (30.2)
None/unknown	27 (62.8)
Ethical awareness	23 (53.5)
Legal guidelines in a hospital evacuation plan	10 (23.3)

#### Staff

All hospitals answered that they have staff recruitment strategies as illustrated in the attached contingency plans and elaborated in free text comments. On-call intrahospital staff was recruited using multiple channels. None of the hospitals, however, had a supplemental surge plan if the incident expanded, and the community staff was not included or mentioned.

#### Stuff

Thirty-six hospitals reported having policies in place for the mobilization of medical devices and ambulances. The protocols included directions for relocating medical devices to the new locations where treatments would be administered. Only four facilities reported sharing equipment with other organizations, including the provincial public health office, non-profit organizations, and local non-medical related organizations. In addition, 14 hospitals reported connecting ambulance services and vehicles to those of other hospitals in the province, which were part of a regional network managed by local health authorities or ambulance operation centers. Out of 36 facilities, only 8 claimed that they operated ambulances independently. Half of the respondents commented that medicines were transported with patients to other facilities for a maximum of 1−3 days.

#### Structure

Twenty-seven hospitals reported conducting treatment areas reconnaissance for potential alternative treatment areas. However, neither evidence nor plans to support their statements regarding space management were presented. Moreover, they expressed that they preferred using routine referral processes to transfer patients to other hospitals than establishing an alternative treatment area.

#### System

Twenty-nine hospitals stated that they prepared systems for disaster responses according to CSCATTT. There were statistically significant associations between having prepared systems and collaboration with police or fire departments (p < 0.05) and between having prepared systems and shared stuff (p < 0.05).

*Command and control* All hospitals claimed to have an incident command system (ICS). Most operated independently except for one hospital that declared a mutual ICS with the local health authorities as a network (Table 2). In addition, most hospitals commented on having plans and various degrees of resource sharing with other public and private organizations.

- Vulnerable group management

Fifteen hospitals stated that they have appropriate guidelines for vulnerable populations. Nevertheless, only two hospitals gave details on the procedures for carrying vulnerable patients using two personnel, and one hospital clarified having emergency sounds and lights for deaf and blind people. Other facilities vaguely described the measures that they claimed were available, and there were no written instructions on the procedures.

- Ethical considerations

Twenty-three hospitals disclosed their ethical awareness and concerns. The viewpoints dwelt on the moral conundrum of leaving critically ill patients behind, as well as equality issues and the difficulty of accessing healthcare during DPHE. Others admitted their negligence in discussing the matters while planning. Nonetheless, one hospital raised concerns about how the public perceives the matter of patient abandonment.

- Legal responsibility

Most hospitals expressed concerns regarding their legal obligations and patient rights, considering the lack of national legal clarity or protection. Furthermore, they anticipated that higher authorities would take responsibility for legal matters and construct proper legislation to safeguard the workforce.

*Safety* Twenty-seven hospitals commented on entrusting police officers to oversee traffic safety, facilitate patients’ transportation, and assist with forensic matters. According to 34 hospitals, the first responders were expected to help handle patients, including triage, first aid, and lifting and transferring, and limit the number of further harms. Ten hospitals stated that they would rely on fire services to deal with fires and other hazardous events.

*Communication* The respondents mentioned both vertical and horizontal communications. The attached plans, according to the ICS, supported the comments. While vertical communications were initiated by either the heads of departments or hospital directors, horizontal communications were performed concurrently from colleague to colleague. The communication channels included personal telephone calls, messaging, social media connections through Line applications, and public announcements. The same communication lines were immediately activated in a second surge during the same incident, if necessary. No issues concerning communication pathways were reported.

*Assessment* The respondents indicated that the incident command group assessed the situation and was responsible for contacting relevant authorities and organizations to obtain necessary data for a mutual assessment.

*Triage* All hospitals had a triage system and emphasized the patients’ prioritizations during an emergency in their comments. Most of them, however, expressed using emergency severity index (ESI) triage during evacuation. Although 28 facilities commented on their familiarity with the reverse triage system, only 6 out of 28 correctly elaborated. Many of them still lacked the necessary knowledge.

*Training* Most hospitals answered that they annually practiced their mass casualty responses or fire evacuation protocols (Table 3). The training details range from functional and tabletop exercises to live simulations. Nevertheless, no respondent mentioned their hospital evacuation exercises or drills, nor did anyone comment on their joint training with other organizations.

**Table 3:** Healthcare worker’s knowledge and training in 43 Thai hospitals

Functions	No. of Hospitals (%)
Triage system in hospital evacuation	
MIMMS triage (sieve/sort)	10 (23.3)
MIMMS triage (start/ESI)	3 (7.0)
ESI	15 (35.7)
Reverse triage	3 (7.0)
In-hospital early warning score	3 (7.0)
Individual triage	4 (9.3)
None/unknown	5 (11.6)
Reverse triage literacy	
Known and occasionally used	13 (31.0)
Known, but have never used	5 (11.6)
Do not know	25 (58.1)
MCI training with other organizations	
Three to four times/year	3 (7.0)
Two times/year	3 (7.0)
One time/year	32 (74.4)
None	3 (7.0)
Unknown	1 (2.3)
Hospital evacuation training (fire response)	
More than one time/year	1 (2.3)
One time/year	31 (72.1)
Less than one time/year	1 (2.3)
None	10 (23.3)

*MCI* Major incidents, *MIMMS* major incident medical management and support, *ESI* emergency severity index

*Transport*

- Internal and ordinary external transportation

According to 29 hospitals, the internal capacities and ordinary external patient transportation were either centrally from networks or locally independently available. Moreover, 14 hospitals described the expectation of additional transportation from nongovernmental organizations to assist patient transfer to other facilities.

- Critical care transportation

Twenty-eight facilities commented on having plans for critical care transportation, which focused on patient prioritizations and placements at proper facilities. Nonetheless, only one hospital elaborated reliable vertical and horizontal intra-hospital transport measures such as a reel or soft stretcher.

### Collaboration

Table 4 represents collaborations for evacuation among hospitals and other organizations.

**Table 4:** Collaborations regarding hospital evacuation in 43 Thai hospitals

Functions	No. of Hospitals (%)
Inter-organization collaboration	
Other hospitals	38 (88.4)
Municipal	30 (69.8)
Police department or fire department	35 (81.4)
First responder	1 (2.3)
Private actors’ collaboration	
Full collaboration	4 (9.3)
Partial collaboration	15 (34.9)
No collaboration	19 (44.2)
No adjacent private actor	3 (7.0)
Unknown	2 (4.7)
Resource management among organizations	
Shared staff	34 (79.1)
Shared stuff	28 (65.1)
No sharing of resources	4 (9.3)
Unknown	3 (7.0)

#### Inter-Organizations

In general, there was scant collaboration both within the healthcare systems, that is, between hospitals, and with other organizations, like police departments, fire departments, non-profit organizations, and private actors. Mutual exercise was also lacking.

#### Inter-Hospitals

Thirty-eight facilities described holding regular meetings to design regional cooperation, coordination, and communication. These meetings involved risk and vulnerability analysis, mitigation, and issuing protocols regarding resource sharing and mutual guidelines. However, the mutual protocols and plans were never rehearsed with joint simulation exercises to ensure feasibility and application.

#### Public Organizations

Most hospitals expressed the awareness of police departments, first responders, and fire departments’ involvement. The respondents described how they expected the parties involved to act including the police to secure the scene and the fire service to contain the fires. However, there were communication inadequacies across facilities both during the preparation phase, when a seamless contingency plan needed to be established and during the response phase, when it was necessary to communicate as the situation progressed.

#### Non-profit Organizations

The respondents asserted that the insufficiencies in all 4S of surge capacity were anticipated to be filled by non-profit organizations such as the Red Cross. Thus, they should be obliged to participate in the planning process and disclose their roles during the preparation phase. They were also expected to supply victims and staff with food, water, shelter, and clothing during the response and recovery phases.

#### Private Actors

Four hospitals reported that private hospitals offered full support for all aspects of mitigation, preparation, formulation of disaster contingency plans, disaster response, and recovery. Another 19 hospitals claimed no collaboration with private actors, and the connections were confined to inter-hospital transportation. Additionally, 4 out of 15 hospitals that partially collaborated with private actors described resources sharing with private non-medical organizations, such as using first-aid appliances from adjacent industries or refueling free gas from gas stations during emergencies.

## Discussion

In this study, Thai hospitals provided credible evidence of independent incident command systems (ICS) and reported annual training. Although the study’s sample size is too small to infer the association between hospital bed capacity and surge planning and collaboration, all hospitals, regardless of bed capacity, exhibited ICS. The results contradicted a study of 1100 US hospitals in 2008 that indicated a correlation between larger bed capacity and safety cooperation and drills (Niska 2008). Nevertheless, the hospital sizes in the US study were different from our study. Of the US study examined hospitals, 60% had less than 100 beds whereas 70% of our participants had capacities of over 300 beds.

Regarding evacuation, our findings demonstrated that hospitals adopted the ICS protocols for evacuation management in some areas, including personnel surges and medicine and device stockpiling, which is consistent with previous studies (Khorram-Manesh et al. 2014; Mace and Sharma 2020). However, the planned resource utilization was limited within the healthcare system and focused on fire escape procedures. The dearth of guidelines exclusive to evacuation may be attributed to the rarity of considerable hospital hazards that necessitate evacuations, despite rising of such threats (CRED and UNDRR 2020). Additionally, the previous responses to major incidents in Thailand showed unfavorable health and socioeconomics outcomes (Wattanawaitunechai et al. 2005; Angthong et al. 2012; Khorram-Manesh et al. 2014), and the findings demonstrate a need to revisit preparedness measures, particularly evacuation plans for all causes, and the use of non-medical resources, such as community resources. Thus, it is plausible to systematically incorporate the FSC concept to establish comprehensive surge planning and a seamless multiagency collaboration (Khorram-Manesh, Phattharapornjaroen, et al. 2021; Yazdani et al. 2021).

### Surge Planning

The surveyed facilities indicated under-developed surge planning and incomplete preparedness, consistent with disaster resilience reports from other countries (King et al. 2016; Ceferino et al. 2020; Hines and Reid 2021), despite the recommendations of surge expansion and information on how to harness the resources and capabilities of local, municipal, and nongovernmental organizations (Gajda 2004; Khorram-Manesh 2020). Moreover, community resources, described in the literature as a FSC, were scarcely addressed, reflecting substantial improvement opportunities. Community resources have an essential role in developing a comprehensive disaster response system, particularly in the hospital evacuation event, which is a complex process, and should be considered in future planning (Khorram-Manesh 2020; Haldane et al. 2021; Phattharapornjaroen et al. 2022b).

#### Staff and Stuff

Most hospitals presented plans and protocols for primary and secondary staff and stuff surges. However, similar to other countries, these capacities were circulated within the healthcare system and were underserved during DPHE (Rojek and Little 2013; Khorram-Manesh et al. 2014; Carli and Telion 2018; Bahrami et al. 2020; Mace and Sharma 2020). Some facilities mentioned their positive experiences of using local resources, but those were only spontaneous actions, and there was no systematic approach. Nevertheless, previous studies exploring additional resources revealed the willingness of medical and non-medical community members with diverse competencies to manage minor injuries and non-disaster-related and uncomplicated medical illnesses. Community facilities also desired to share first aid and necessary medical devices (Glantz et al. 2020; Khorram-Manesh et al. 2020). Community’s leverages are invaluable during a hospital evacuation but necessitate proper education and continuous exercise.

#### Structures

Although most hospitals would prefer to transfer their patients to other healthcare facilities, some emergency scenarios still require field hospitals, shelter-in-place, or other local facilities because of damaged infrastructures (King et al. 2016; Carli and Telion 2018; Hines and Reid 2021). In these circumstances, the flexible treatment spaces may be augmented using local areas (Glantz et al. 2020; Khorram-Manesh 2020; Phattharapornjaroen et al. 2022b), particularly when hospital infrastructures and local transportation connections are disrupted, and immediate adjacent resources as well as spaces belonging to other agencies or organizations are needed. Such flexibility can be achieved through productive collaboration between various authorities in peacetime to be ready for emergencies and disasters. For example, when hospitals and residential areas were bombarded during the ongoing conflict in Ukrainian, roads were destroyed and patients and healthcare personnel had to seek shelters in local buildings, and food and water were supplied by neighborhood restaurants (Reed 2022). This approach requires thorough familiarity with community resources, which was absent among the participants in this study. Information regarding the concept of FSC and educational initiatives would be needed to increase staff and community awareness about local resources and their feasibility of use.

#### System

Most hospitals reported minimal concern about ethical and legal issues during disasters, and some did not address these issues. One reason for such unawareness could be a lack of previous lawsuits against healthcare providers for legal and ethical violations during disasters and emergencies. Another reason might be the lack of discussion and awareness of such issues among the authorities and populations in society. Nevertheless, the participants expected local health authorities to consider and enact legislation regulating patients’ treatment during crises, mainly when the only option during an evacuation is to leave someone behind with or without knowledge and perception of reverse triage. Currently, there are neither laws nor rules in Thailand to protect healthcare personnel in charge of tactical and strategic medical decision making during crises. However, the United States National Academy of Medicine has suggested striking a balance between the standard of care and the requirements of the larger community (Hick et al. 2020). The FSC concept bases operational changes on the incident command and informed decision-making processes, allowing for ethical and legal resource management (Gostin et al. 2020; Hick et al. 2020).

The reverse triage system has been introduced for decades (Kelen et al. 2009; Pollaris and Sabbe 2016; Caramello et al. 2019). It provides the ability to increase hospital surge capacities based on the utilitarian concept (Gillon 1985) (that is, the greatest good for the greatest number), which is also acceptable during disaster responses. The concept became more challenging during the Covid-19 pandemic when the demand for ventilators and hospital admissions significantly increased (Savulescu et al. 2020). However, the findings in this study showed a considerable knowledge gap for reverse triage and its related ethical and legal concerns. Thus, the reverse triage system and related regulations should be constructed to facilitate healthcare performance in the best interest of patients with support from the health authorities.

With limited logistical resources, some hospitals expressed their concerns about disproportionate resource consumption in critical care transportation (Einav et al. 2014; Hick et al. 2014; Hugelius et al. 2020). Critical patients’ outcomes may be affected by various transportation challenges, including pre-transfer procedures (initial stabilization and damage control procedures), during transfer processes (staff skills, available medical devices), and finally, the readiness of receiving facilities (King et al. 2014). The findings of this study were consistent with those of other research, revealing a shortcoming in transportation arrangements and a lack of augmentation protocols for both horizontal and vertical transportation (Einav et al. 2014; Hicks and Glick 2015), underscoring the urgent need for hospitals to develop practical transportation management guidelines. Utilizing a solid triage system, including a reverse triage system, to critically examine all patients requiring critical transfer to a hospital, is essential to relieving transport complications.

### Collaborations

The results showed a certain degree of collaboration between hospitals and other organizations, such as task coordination to refer patients and sharing information through communication with the local health authority. Moreover, the statistically significant associations between the available system that participating facilities claimed to have in surge capacity and police or fire departments and between the available system and shared stuff indicate the directions that hospitals with established systems would have organized collaboration, including shared stuff, and with police or fire departments. However, collaboration requires cooperation, coordination, and communication, and no hospitals showed complete collaboration, which also emphasized the need for preparedness and training (Gajda 2004; Lozano et al. 2021; Phattharapornjaroen et al. 2022a, 2022b).

In summary, the findings of this study accentuated the shortcomings of hospital evacuation preparedness in terms of comprehensive surge planning including staff, stuff, and structure capacities and capabilities to create effective systems, seamless multiagency collaboration, and opportunities for community engagement. Previous studies have demonstrated the value of realistic and practical exercises and training in improving disaster responses (Sammut et al. 2001; Berlin and Carlström 2015; Khorram-Manesh et al. 2015; Kelly et al. 2021). Moreover, they facilitate the identification of contingency plan gaps and potential technical and strategic challenges. One solution for these challenges is providing training and exercises, which involve individuals, communities, organizations, and authorities as well as further conduction of qualitative research exploring effective training procedures using community facilities. Although much easier than a full evacuation, horizontal as well as vertical evacuations within the hospital may also cause difficulties that should be explored through education and full-scale training to find the small issues that will jeopardize the entire operation.

## Limitations

Although the survey with an online approach was considered the most suitable method during the current pandemic due to the in-person contact limitation policy, a face-to-face interview could be a more straightforward way to achieve the highest degree of responses. The low response rate in this study may reflect the negligence of the evacuation preparedness. However, a nationally issued and supported survey may increase the response rate in future studies. Additionally, selection bias might have occurred in this study, as in all survey studies since the facilities that were more interested or prepared were more likely to participate. However, this study’s response rate met the expectation given by past research in a similar design (Aerny-Perreten et al. 2015; Ball 2019). Another significant limitation is that the data were collected when the pandemic halted most hospital routine preparation. Therefore, information about some items, such as the training, may include recall and selection bias.

## Conclusion

Hospitals are vulnerable to internal and external hazards, which may result in an evacuation. Such evacuation needs internal and external resources to assure the safety of affected patients. This study, to the best of our knowledge, is the first study assessing hospital evacuation preparedness in Thailand and elsewhere using the theoretical and conceptual frameworks for the FSC. Focusing on surge capacity’s four vital elements and collaborative factors encompassing CSCATTT, the study’s findings indicate a gap in hospital evacuation procedures, particularly triage and logistics, and inadequacies in surge planning and multiagency collaboration, in which the FSC implementation is plausible. Although using community resources may be beneficial during a disaster, the measures for concept integration into hospital evacuation are still challenging.

Nevertheless, pragmatic research exploring planning for community engagement according to the flexible surge capacity to build a concrete hospital evacuation plan would enhance hospital readiness and its generalizations. The latter needs to be tested in simulation exercises. In conclusion, the concept of FSC and its collaborative tool seem valuable for evaluating hospital evacuation preparedness and creating educational initiatives to enhance such preparedness.

## Footnotes

1 The datasets generated and/or analyzed during this study and the study tool are available in the Harvard Dataverse repository at https://doi.org/10.7910/DVN/HW0F5Q.
